# Cottage Hospitals and Medical Fees

**Published:** 1897-01-02

**Authors:** 


### Editor's Letter-Box.

[Our Correspondents are reminded that prolixity is a great bar to publication, and that brevity of style and conciseness of statement greatly facilitate early insertion.]

### COTTAGE HOSPITALS AND MEDICAL FEES.

In regard to the case mentioned in The Hospital on December 12th, General Maclean, honorary secretary of the Victoria Cottage Hospital, Wimborne, writes: "The case was one of emergency, the patient admitted on the application by telegram of a well-known medical man—from the house of the patient's employer—who sent by post a ticket of admission, which was submitted to the visitors at their usual weekly meeting, when the retention of the patient was approved of, and the terms to be paid to the hospital were settled, and the employer made aware of the charge. A second ticket of admission at the end of fourteen days was received from the employer, but notice sent that the patient would have to pay the charge for maintenance in the hospital herself, as her term of service with Mrs. Drake had terminated. This was duly put before the visitors, who made a reduction in the charge from the account usually claimed for domestic servants coming from the house of their employers. The committee declined to admit that a domestic servant came within their rules as to the "working class," to whom the medical staff offered their attendance gratuitously."

*** This exactly bears out what we said. The patient was admitted as an emergency, and until a special charge was arranged by the visitors the medical officer could not make any charge. The error—if there was any error—clearly lay in not acquainting the employer with the fact that if the patient were to be admitted on special terms these would not include the charge for medical attendance.—Ed. *T. H.*

